# Psychological variables associated with quality of life in patients with head and neck cancer: the role of body image distress

**DOI:** 10.1007/s00520-022-07334-6

**Published:** 2022-08-23

**Authors:** Silvia Cerea, Maria Sansoni, Giovanni Scarzello, Elena Groff, Marta Ghisi

**Affiliations:** 1 Department of General Psychology, University of Padova, Via Venezia, 8 35131 Padua, Italy; 2 Department of Biomedical Sciences, University of Padova, Padua, Italy; 3 Department of Psychology, Catholic University of Sacred Heart, Milan, Italy; 4 Veneto Institute of Oncology (IOV), Padua, Italy; 5 U.O.C. Hospital Psychology, University-Hospital of Padova, Padua, Italy

**Keywords:** Head and neck cancer, Body image, Quality of life, Psychological variables, Pain, Gender differences

## Abstract

**Objective:**

The aim of this cross-sectional study was to explore the relationship between quality of life (QoL) and body image distress in patients with head and neck cancer (HNC), considering relevant psychological variables (i.e., coping strategies, social anxiety symptoms, self-esteem, intolerance of uncertainty, pain, and distress). We also aimed to explore gender differences in patients with HNC in terms of relevant psychological variables in HNC.

**Methods:**

Fifty-one HNC patients (37 males and 14 females) completed self-report questionnaires to assess body image distress, physical and mental QoL, and relevant psychological variables in HNC (coping strategies, social anxiety symptoms, self-esteem, intolerance of uncertainty, pain, and distress) before undergoing treatment. Pearson’s correlations and four-step hierarchical regressions were performed to assess the relationship between body image distress, QoL, and the abovementioned psychological variables, while one-way analyses of variance and one-way analysis of covariance were employed to assess gender differences.

**Results:**

Physical QoL was associated with body image distress above and beyond disease duration, distress, coping strategies, pain, mental QoL, and self-esteem, while mental QoL was associated with pain above and beyond distress, coping strategies, physical QoL, self-esteem, and body image distress. Concerning gender differences, females scored higher than males on most of the explored psychological variables, except for physical QoL and intolerance of uncertainty, and showed lower mental QoL and self-esteem than males.

**Conclusion:**

Body image distress and pain emerged as negatively associated with QoL, and almost all the explored psychological variables differed among genders. Psychological interventions targeting body image distress and pain should be promoted in patients with HNC to increase their QoL, while keeping gender differences in mind.

**Supplementary Information:**

The online version contains supplementary material available at 10.1007/s00520-022-07334-6.

## Introduction

Body image is the internal representation that individuals have of their own body and physical appearance [1]. In recent years, body image has received increased attention in medical settings, particularly in those affecting physical appearance such as head and neck cancer (HNC; [2]). Indeed, the disease and its treatment generate serious alterations of physical appearance (e.g., asymmetry, altered muscle movement, and scarring) [3, 4]. HNC-related physical changes are particularly upsetting and impairing for patients because they occur in highly visible and socially significant parts of the body (e.g., face), making it extremely hard for the individuals to conceal them [2, 3]. For such reasons, patients with HNC are at high risk of experiencing body image distress, which represents one of the most distressing psychosocial issues in this population [2].

Body image distress due to HNC is associated with a variety of life changes (e.g., psychological distress, anxiety, sexual dysfunction, and social isolation and withdrawal) [2] and may negatively impact quality of life (QoL). However, few studies have examined the relationship between body image distress and QoL in patients with HNC, possibly because body image is considered an aspect of QoL in this population, and it has been frequently assessed with subscales within the QoL instruments [5]. Furthermore, the majority of the studies exploring the relationship between body image distress and QoL have not considered important psychological variables related to QoL in patients with HNC (e.g., coping strategies, social anxiety symptoms, self-esteem, intolerance of uncertainty, and presence of pain), or have considered only some of these variables (e.g., [6–8]).

In accordance with the conceptual framework of Rhoten and colleagues [3], patients’ characteristics, social factors, and environmental factors are associated with body image distress and, in turn, with patients’ overall QoL. This conceptual framework is also supported by two recent systematic reviews [9, 10] showing that psychological variables are associated with HNC diagnosis, treatment, and recovery. For instance, patients’ ability to respond to and manage internal and external demands (i.e., coping strategies) related to HNC has a strong impact on QoL [8]. Indeed, one of the biggest challenges of these patients may be their (in)ability to cope with changes in their physical appearance. The fear of a negative evaluation from others (i.e., the core feature of social anxiety symptoms) represents another psychological variable that may impact patients’ adjustment to the cancer journey. In fact, due to the increased likelihood of disfigurements caused by the HNC disease and its treatment and the implications of visible differences in social interactions, patients with HNC frequently experience poor social self-efficacy and social isolation [11, 12]. This predisposes them to the development of social anxiety symptoms [13]. In addition to coping strategies and social anxiety symptoms, self-esteem may also play a role in HNC patients’ QoL, as emerged in previous studies (e.g., [14]), showing that low self-esteem was associated with poor QoL and psychological distress in patients with HNC. Intolerance of uncertainty (IU) may also be relevant in individuals with cancer, because frequently both disease progression and prognoses are unknown [15, 16], with detrimental effects on patients’ QoL [17]. Therefore, being able to manage uncertainty may be critical for the QoL of patients with cancer, and recent evidence has suggested that IU may be associated with poor QoL in patients with different cancer condition [6, 18], despite no previous studies have assessed IU in patients with HNC. Lastly, in HNC, pain represents a major issue before, during, and after treatments and may persist for years [7], negatively impacting QoL [7, 19].

Gender differences pertaining to body image distress and other relevant psychological variables before undergoing HNC treatment deserve more attention as well. Indeed, few studies are available on this topic and inconsistent results emerged from studies conducted after HNC treatment [2]. The study conducted by Fingeret and colleagues [4] on newly diagnosed HNC patients found no gender differences in body image concerns. However, some authors speculated that it is harder for men to disguise an altered physical appearance since they tend not to use makeup, scarves, or other accessories which could hide the disfigured area [20]. A deeper investigation of gender differences before HNC treatment is crucial to deeply understand the unique experience of body image distress in HNC populations.

The first aim of this cross-sectional study was to investigate the relationship between QoL and body image distress in patients with HNC. To explore this relationship, we focused on the preoperative period to obtain estimates of body image distress associated with the anticipation of a potentially disfiguring treatment [4]. Previous research supports the influence of preoperative expectations/anticipation of disfigurative HNC treatment on distress, anxiety, coping effectiveness, and post-operative satisfaction [21–24]. Moreover, patients with HNC reported elevated body image distress even before treatment [25], due to the HNC illness itself (i.e., often impacting physical appearance). Given the impact of HNC disease on physical appearance [25], we expected that body image distress will be negatively associated to both physical and mental QoL, over and above coping strategies, social anxiety symptoms, self-esteem, IU, and presence of pain.

To shed light on gender differences before HNC treatment, the second aim of the study was to explore gender differences in body image distress, QoL, and associated psychological variables (i.e., coping strategies, social anxiety symptoms, self-esteem, IU, presence of pain, and general distress). In accordance with evidence points to higher rates of certain psychological conditions in women, especially related to body image [26], we hypothesize to find higher levels of body image distress, social anxiety symptoms, IU, and general distress, as well as lower QoL, self-esteem, and adaptive coping strategies, in women compared to men.

To our knowledge, no previous studies have considered all these relevant psychological variables to deepen the relationship between QoL and body image distress in patients with HNC. A better understanding of the relationship between body image, physical and mental QoL, and relevant psychological variables in HNC will allow clinicians to better understand how patients react to disfigurement and dysfunctions related to HNC and its treatment. Findings of this cross-sectional study may therefore have important implications for early identification and treatment of body image distress in patients with HNC that can potentially improve the QoL of these patients [3].

## Methods

### Study design

This is a cross-sectional study: participants completed self-report questionnaires before undergoing a medical treatment for HNC (no other assessments occurred).

### Participants

Patients with HNC who were about to receive a medical treatment approximately in a month (surgery, radiotherapy, chemotherapy, or concomitant radiotherapy and chemotherapy) were asked to take part in the study. Eligible patients were identified during multidisciplinary HNC visits at the Radiotherapy Unit of the Veneto Institute of Oncology (IOV) in Padua (Italy). Eligible criteria included patients who (1) were older than 18 years, (2) were diagnosed with a HNC, (3) were about to receive a medical treatment for HNC (all tumor sites, all treatment modalities), (4) were not receiving medical treatments for other cancer diseases at the time of the research, and (5) were competent to provide informed consent. No restriction was placed on the type of treatment participants received/tumor sites for inclusion in the study. The only exclusion criterion was the presence of a benign neoplasm localized on the head and neck anatomical district (e.g., parotid). Based on such criteria, 51 patients (37 males and 14 females) were considered eligible and were enrolled for the study (Table 1). In terms of response rate, only one patient considered eligible did not agree to take part in the study, a 50-year-old female with a tumor localized in the oral cavity.^1^

**Table 1:** Patients’ sociodemographic and medical characteristics (*n* = 51)

	*n* (%) or M (SD)
Age	63.14 (11.63)
Gender	
Male	37 (72.5)
Female	14 (27.5)
Education (in years)	10.57 (3.61)
Marital status	
Single/never married	8 (15.7)
Married or living with S/O	37 (72.5)
Divorced/separated	5 (9.8)
Widowed	1 (2.0)
Occupation	
Full-time employed	14 (27.5)
Part-time employed	1 (2.0)
Housewife	3 (5.9)
Unemployed	4 (7.8)
Retired	22 (43.1)
Not able to work for disability	1 (2.0)
Other	6 (11.8)
Time from the diagnosis (in months)	17.66 (31.38)
Tumor localization	
Salivary glands	4 (7.8)
Pharynx	25 (51.0)
Oral cavity	12 (23.5)
Larynx	6 (11.8)
Paranasal sinus and nasal cavity	2 (3.9)
Skin	2 (3.9)
Tumor stage	
I	5 (9.8)
II	4 (7.8)
III	12 (23.5)
IV	24 (47.1)
Not possible to specify	6 (11.8)
Previous treatments for HNC	
Surgical	25 (49.0)
Not-surgical (RT, CT, CRT)	12 (23.5)
Ongoing treatments for HNC (focus of the current study)	
Surgery	12 (23.5)
RT	18 (35.3)
CT	9 (17.6)
CRT	12 (23.5)

*Note. M*, mean; *SD*, standard deviation; *S/O*, significant other; *HNC*, head and neck cancer; *RT*, radiotherapy; *CT*, chemotherapy; *CRT*, chemotherapy and radiotherapy

### Recruitment procedures

Patients with HNC were recruited between February 2019 and February 2020. A licensed clinical psychologist with extensive experience managing pyscho-oncologic concerns in patients with HNC recruited patients during multidisciplinary HNC visits where patients received the cancer diagnosis and were offered a treatment. The clinical psychologist explained the study aims and patients were asked to complete a survey pertaining to the relationship between body image and physical and psychological well-being. No other assessments took place. Interested and eligible patients gave their written informed consent and completed self-report questionnaires before receiving treatment for HNC. Patients with HNC who accepted to participate in the study were offered two different modalities to complete the survey: in-person, at the Radiotherapy Unit of the Veneto Institute of Oncology (IOV), or at home. Two modalities of compilation were offered since most patients were willing to participate in the study but were distress after the HNC visit. When patients preferred the in-person modality, they completed self-report questionnaires in a quiet room at the IOV, specifically arranged for the self-report questionnaires compilation; the clinical psychologist was available to answer any inquiry during the filling process. The time for the compilation was approximately 40 min. When patients preferred the at-home modality, the clinical psychologist provided self-report questionnaires and a sealing envelop and instructed patients to complete the questionnaires at home and to take them back in the sealing envelop the day of the next HNC visit (approximately 2 weeks after). Patients did not receive any compensation for their participation. The study was conducted in accordance with the Declaration of Helsinki and approved by the Ethics Committee of the Psychological Research of the University of Padua.

### Measures

S*ocio-demographic information form:* employed to assess socio-demographic information of participants such as sex (biological, assigned at birth; “What is your sex assigned at birth?”), gender (the internal/psychological sense of self, regardless of what sex a person was assigned at birth; “What is your current gender identity?”), age, education, and self-reported psychological disorders.

*Personal medical history:* information about participants’ medical history was collected through the electronic medical record of each patient, including cancer history (presence of recurrence and previous treatments for HNC), time of diagnosis, stage and localization of disease, and medications.

*Body Image Scale* (BIS; [27, 28]): self-report questionnaire aimed at investigating body image distress in patients with cancer. The BIS measures emotional, cognitive, and behavioral components of body image through 10 items on a 4-point Likert scale (from 1 = “never” to 4 = “very”). Higher values correspond to greater body image distress. The Italian version of the BIS showed good psychometric properties (Cronbach’s α = 0.92). In the current study, the BIS showed good internal consistency (Cronbach’s α = 0.93).

*Short Form-12 Health Survey* (SF-12; [29; 30]): a brief version of the SF-36 health survey. The SF-12 evaluates eight dimensions related to an individual’s life that can be influenced by the presence of a disease. Answers can be provided through dichotomous yes/no answers, or through items on a 3- or a 5-point Likert scale. In addition to the eight dimensions, the SF-12 produces two summary scores evaluating physical and mental health. Higher scores are associated with higher QoL. The Italian version of the SF-12 showed good content, construct, and criterion validity [31]. For the purposes of the study, we considered only the Physical Component Score (PCS) and the Mental Component Score (MCS).

*Rosenberg Self-Esteem Scale* (RSES; [32; 33]): measure made up of 10 items rated on a 4-point Likert scale (from 1 = “strongly disagree” to 4 = “strongly agree”) assessing global self-esteem, with higher scores indicating greater self-esteem. The Italian version of the RSES showed good psychometric properties: its internal consistency was α = 0.84 [33]. In the current study, the RSES showed adequate internal consistency (Cronbach’s α = 0.72).

*Intolerance of Uncertainty Scale-12* (IUS-12; [34; 35]): 12-item self-report measure evaluating the tendency to find uncertainty upsetting and distressing. Individuals are asked to rate the extent to which each statement applies to themselves on a 5-point Likert scale (from 1 = “not at all like me” to 5 = “entirely like me”), with higher scores indicating greater IU. The IUS-12 demonstrated excellent internal consistency (Cronbach’s α = 0.80), convergent, and discriminant validity in its Italian version [35]. In the current sample, internal consistency was good (α = 0.87).

*Social Interaction Anxiety Scale* (SIAS; [36; 37]): 19-item self-report questionnaire designed to assess social interaction anxiety on a 5-point Likert scale (from 0 = “Not at all” to 4 = “Extremely”), with higher scores indicating greater social anxiety symptoms. The Italian version of the SIAS proved to be highly reliable (Cronbach’s α = 0.86) [37]. In our samples, the SIAS showed good internal consistency (Cronbach’s α = 0.84).

*Coping Response Inventory Adult Form* (CRI-Adult; [38; 39]): self-report questionnaire made up of 48 items assessing approach (logical analysis, positive reappraisal, guidance/support seeking, and problem solving) and avoidance coping (cognitive avoidance, resigned acceptance, alternative rewards, and emotional discharge). Each subscale is composed of six items rated on a 4-point Likert-type scale (0 = “Not at all”; 3 = “Fairly often”). Participants had to think specifically about how they cope with the diagnosis of HNC when replying to the items of the questionnaire. The administration of the Italian version of the CRI-Adult showed internal consistency values ranging from α = 0.58 to α = 0.68 for the approach scales, and ranging from α = 0.57 to α = 0.66 for the avoidance scales [39]. In the present sample, α coefficients for the approach scales ranged from α = 0.60 to α = 0.72, while α values for the avoidance scales ranged from α = 0.60 to α = 0.65.

*Depression Anxiety Stress Scale-21* (DASS-21; [40; 41]): 21-item self-report questionnaire assessing depression, anxiety, and stress on a 4-point Likert scale (from 0 = “did not apply to me at all” to 3 = “applied to me very much”), with higher scores indicating greater distress. The Italian version of the DASS-21 proved to be highly reliable (from α = 0.74 to α = 0.90 [41]. Findings of the Italian version suggested that the use of the total score, measuring a “general distress” factor, could be more appropriate than calculating the three subscales separately [41]. Therefore, we focused only on the total score of the scale (i.e., general distress). In the current study, the DASS-21 total score showed good internal consistency (Cronbach’s α = 0.94).

*Brief pain inventory* (BPI; [42; 43]): 15-item self-report questionnaire designed to measure pain intensity and impact of pain on daily functioning. The BPI allows to calculate a total score and two specific scores (pain severity and pain interference) on a 10-point Likert Scale (0 = “no pain”/ “does not interfere”; 10 = “pain as bad as you can imagine”/ “interferes completely”), with higher scores indicating higher pain severity and interference. The Italian version of the BPI showed good psychometric properties [43]. For the purposes of the study, we focused only on the total score of the questionnaire. In the current study, the BPI showed good internal consistency (Cronbach’s α = 0.90).

### Statistical analyses

To investigate the relationship between body image distress (BIS) and physical (PCS-SF-12) and mental (MCS-SF-12) QoL, preliminary Pearson’s correlation analyses were performed. Correlations between age, disease duration, and self-report measures (i.e., scores obtained at the SF-12, BIS, RSES, IUS, SIAS, CRI-Adult, BPI, and DASS-21) were performed on the whole sample to identify variables to include in the regression model (see Supplementary Materials). Based on results emerged from correlational findings (i.e., only variables significantly correlated with the dependent variables were included in regression models), two multiple hierarchical regression models were performed. Pertaining to physical QoL (as measured by the PCS of the SF-12), a four-step multiple hierarchical regression analysis was performed, wherein the physical QoL (PCS) was included as dependent variable. Both time from diagnosis and DASS-21 total score were included in the first block to control for disease duration and general distress. Appropriate coping strategies (i.e., positive reappraisal and alternative rewards subscales of the CRI-Adult) were included in the second block, whereas variables related to mental QoL and pain (i.e., MCS and BPI total score) were included in the third block. Finally, psychological variables (i.e., RSES and BIS) were included in the fourth block. Also pertaining to mental QoL (as measured by the MCS of the SF-12), a four-step multiple hierarchical regression analysis was performed, wherein the mental QoL (MCS) was included as a dependent variable. The DASS-21 total score was included in the first block to control for general distress; coping strategies (i.e., logical analysis, resigned acceptance, and emotional discharge subscales of the CRI-Adult) were included in the second block, whereas variables related to physical QoL and pain (i.e., PCS and BPI total score) were included the third block. Then, psychological variables (i.e., RSES and BIS) were entered in the fourth block. The sequence of the 4 blocks of variables was driven by our specific research questions in conjunction with a theory-driven approach: we were interested in investigating the relationship between body image distress and QoL above and beyond relevant variables in HNC (HNC-related variables, general distress, coping strategies, physical/mental QoL, presence of pain, and psychological variables). Therefore, we statistically “control” for certain variables, to see whether adding variables significantly improved the model’s ability to account for physical/mental QoL. In the final/full model, it makes no difference as to when a given independent variable was entered; the estimated regression coefficients are conditional based on all other independent variables and the relationships in our data set, and not order of entry. We have indeed changed the sequence of the 4 blocks and tested both regression models: no differences between regression models emerged (i.e., body image distress emerged as associated with physical QoL and the presence of pain emerged as associated with mental QoL). To evaluate the final model fit, we employed the adjusted R squared (% of variance explained). All assumptions for multiple regression analysis were met.

To assess gender differences on socio-demographics (i.e., age and education), HNC-related variables, psychological variables, Chi-squared analyses (χ2), one-way analyses of variance (ANOVAs), and one-way analysis of covariance (ANCOVA)^2^ were performed. Before conducting these statistical analyses, we checked the normality of the distribution of self-report measures. Normality was not met for scores obtained at the SIAS, BIS, BPI, CRI-Adult guidance/support seeking, and CRI-Adult cognitive avoidance. However, analyses with the non-parametric Mann–Whitney test and the Rho Spearman correlation coefficient gave similar results to the ANOVA, ANCOVA, and Pearson’s, so we report on the latter here. Bonferroni correction was applied to the CRI-Adult and the SF-12 scores, revealing a significant *p* value of 0.006 for the CRI-Adult questionnaire and of 0.025 for the SF-12. Partial Eta Squared (*η*_*p*_^2^) values were reported to evaluate the magnitude of effects. Cohen [44] has provided benchmarks to define small (*η*^2^ = 0.01), medium (*η*^2^ = 0.06), and large (*η*^2^ = 0.14) effects when partial eta squared are computed.

A post-hoc analysis of the sample size was established using the G*Power 3.1 software [45]. The variables of the multiple hierarchical regression were considered: disease duration, DASS-21 total score, CRI-Adult logical analysis, CRI-Adult alternative rewards, CRI-Adult emotional discharge, CRI-Adult resigned acceptance, MCS, PCS, BPI total score, BIS total score, and RSES. A total sample of 51 patients allows to achieve a power of 0.81 in reliably detecting a one-tailed effect (f^2^) of 0.13, with a type I error of 0.05.

Less than 0.5% of the total dataset was missing. Given the minimal missing data, these were replaced using the mean replacement method (i.e., replacement using the mean of valid surrounding values).

All statistical analyses were conducted using IBM SPSS statistics [46], version 26, and the G*Power 3.1 software [45].

## Results

### Psychological variables associated with physical QoL

The overall model explained 37.4% (adjusted R square) of the variance in physical QoL (PCS). Disease duration and general distress (DASS-21) were entered in the first step, but were not significantly associated with physical QoL (PCS) (*F*_(2,40)_ = 1.81, *p* = 0.18). The inclusion of coping strategies in the second step of the model did not explain an additional variation (13.6%) in physical QoL (PCS) (*F* change = 3.14; *p* = 0.06), despite alternative reward coping strategy (CRI-Adult) emerged as significant (*p* = 0.04) (i.e., alternative reward strategy emerged as significant, but the general 2nd step was not). The inclusion of mental QoL (MCS) and pain (BPI) in the third step of the model did not explain an additional variation (10.3%) in physical QoL (PCS) (*F* change = 2.60; *p* = 0.09), despite the step emerged as significant (*p* = 0.03). Finally, the inclusion of self-esteem (RSES) and body image distress (BIS) explained an additional 17.3% of the variation in QoL (PCS) (*F* change = 5.53; *p* = 0.01). Results showed that body image distress (BIS) was the only variable significantly associated (negatively) with physical health (PCS), whereas all the other variables were not (Table 2).

**Table 2:** Psychological variables associated with physical health

Variables		B	SE	β	t	*p*	F	df
**Step 1**							1.81	2,49
	Constant	44.85	1.97		22.78	*p* < 0.001		
	Disease duration	− 0.05	0.04	− 0.22	− 1.38	0.17		
	General distress (DASS-21)	− 0.09	0.11	− 0.14	− 0.87	0.39		
**Step 2**							2.85	4,49
	Constant	42.24	3.46		12.22	*p* < 0.001		
	Disease duration	− 0.05	0.04	− 0.23	− 1.48	0.15		
	General distress (DASS-21)	− 0.04	0.13	− 0.06	− 0.32	0.75		
	Resigned acceptance (CRI-Adult)	− 0.27	0.41	− 0.12	− 0.65	0.52		
	Alternative rewards (CRI-Adult)	0.64	0.28	0.34	2.27	0.03		
**Step 3**							2.74*	6,49
	Constant	37.56	7.72		5.16	*p* < 0.001		
	Disease duration	− 0.05	0.03	− 0.22	− 1.46	0.15		
	General distress (DASS-21)	0.02	0.14	0.03	0.17	0.87		
	Resigned acceptance (CRI-Adult)	0.03	0.42	0.01	0.08	0.94		
	Alternative rewards (CRI-Adult)	0.57	0.27	0.30	2.09	0.04		
	Pain (BPI)	− 0.13	0.08	− 0.29	− 1.62	0.11		
	Mental health (MCS)	0.08	0.12	0.14	0.68	0.50		
**Step 4**							3.98*	8,49
	Constant	27.67	11.66		2.37	0.02		
	Disease duration	− 0.04	0.03	− 0.18	− 1.26	0.22		
	General distress (DASS-21)	0.12	0.12	0.17	0.96	0.34		
	Resigned acceptance (CRI-Adult)	0.22	0.38	0.10	0.58	0.56		
	Alternative rewards (CRI-Adult)	0.41	0.25	0.22	1.66	0.11		
	Pain (BPI)	− 0.15	0.07	− 0.32	− 1.99	0.06		
	Mental health (MCS)	0.02	0.11	0.04	0.21	0.83		
	Body image distress (BIS)	− 0.31	0.14	− 0.37	− 2.19	0.04		
	Self-esteem (RSES)	0.38	0.28	0.21	1.37	0.18		

*Note.* DV: *PCS*, physical component score; * *p* < 0.05; ** *p* < 0.001; *SE*, standard error; *df*, degrees of freedom; *DASS-21*, Depression Anxiety Stress Scale-21; *CRI-Adult*, Coping Responses Inventory-Adult Form; *BPI*, Brief Pain Inventory; *MCS*, Mental Component Score; *BIS*, Body Image Scale; *RSES*, Rosenberg Self-Esteem Scale

### Psychological variables associated with mental QoL

The overall model explained 42.4% (adjusted R square) of the variance in mental QoL (MCS). General distress (DASS-21), entered in the first step of the regression model, emerged as significantly associated (negatively) with mental QoL (MCS) (*F*_(1,39)_ = 18.95, *p* < 0.001). The inclusion of coping strategies in the second step did not significantly explain an additional variation (4.1%) in mental QoL (MCS) (*F* change = 0.77; *p* = 0.52). Physical QoL (PCS) and pain (BPI), entered in the third step of the regression model, significantly accounted for mental QoL (MCS), *F* change = 5.48; *p* = 0.01, explaining an additional 15.6% of the variation. Finally, the inclusion of self-esteem (RSES) and body image distress (BIS) did not significantly explain an additional variation (1.2%) in mental QoL (MCS) (*F* change = 0.39; *p* = 0.68). Results showed that pain (BPI) was the only variable significantly associated (negatively) with mental QoL (MCS), whereas all the other variables were not (Table 3).

**Table 3:** Psychological variables associated with mental health

Variables		B	SE	β	t	*p*	F	df
**Step 1**							18.95**	1,49
	Constant	53.51	2.81		19.06	*p* < 0.001		
	General distress (DASS-21)	− 0.65	0.15	− 0.58	− 4.35	*p* < 0.001		
**Step 2**							5.23**	4,49
	Constant	57.04	4.28		13.33	*p* < 0.001		
	General distress (DASS-21)	− 0.43	0.21	− 0.38	− 2.04	0.05		
	Logical analysis (CRI-Adult)	0.12	0.45	0.04	0.26	0.80		
	Resigned acceptance (CRI-Adult)	− 0.55	0.66	− 0.15	− 0.84	0.41		
	Emotional discharge (CRI-Adult)	− 0.75	0.73	− 0.19	− 1.04	0.31		
**Step 3**							6.21**	6,49
	Constant	48.72	11.04		4.41	*p* < 0.001		
	General distress (DASS-21)	− 0.38	0.19	− 0.33	− 1.99	0.05		
	Logical analysis (CRI-Adult)	0.25	0.41	0.09	0.61	0.55		
	Resigned acceptance (CRI-Adult)	0.10	0.62	0.03	0.16	0.87		
	Emotional discharge (CRI-Adult)	− 0.86	0.65	− 0.22	− 1.32	0.19		
	Pain (BPI)	− 0.31	0.11	− 0.41	− 2.77	0.01		
	Physical health (PCS)	0.14	0.23	0.09	0.64	0.53		
**Step 4**							4.58**	8,49
	Constant	57.69	18.27		3.16	0.004		
	General distress (DASS-21)	− 0.33	0.20	− 0.29	− 1.62	0.11		
	Logical analysis (CRI-Adult)	0.20	0.42	0.07	0.47	0.64		
	Resigned acceptance (CRI-Adult)	0.21	0.65	0.06	0.33	0.75		
	Emotional discharge (CRI-Adult)	− 0.91	0.68	− 0.23	− 1.34	0.19		
	Pain (BPI)	− 0.31	0.11	− 0.42	− 2.72	0.01		
	Physical health (PCS)	0.06	0.27	0.03	0.21	0.83		
	Body image distress (BIS)	− 0.14	0.44	− 0.05	− 0.32	0.75		
	Self-esteem (RSES)	− 0.21	0.24	− 0.15	− 0.88	0.39		

*Note.* DV: *MCS*, mental component score; * *p* < 0.05; ** *p* < 0.001; *SE*, standard error; *df*, degrees of freedom; *DASS-21*, Depression Anxiety Stress Scale-21; *CRI-Adult*, Coping Responses Inventory-Adult Form; *BPI*, Brief Pain Inventory; *PCS*, Physical Component Score; *BIS*, Body Image Scale; *RSES*, Rosenberg Self-Esteem Scale

### Gender differences in age, years of education, HNC-related variables, QoL, and psychological variables relevant in HNC

No significant differences between genders with respect to years of education emerged (*p* = 0.40), while age differed: males were older than females (*p* = 0.02). In terms of HNC-related variables, no gender differences emerged (all *ps* > 0.05).

With respect to psychological variables, significant differences between genders emerged: females scored higher than males on body image distress (BIS), social anxiety symptoms (SIAS), general distress (DASS-21), CRI-Adult emotional discharge, and pain (BPI) and scored lower than males on mental QoL (MCS) and self-esteem (RSES). No significant differences emerged with respect to physical QoL (PCS), intolerance of uncertainty (IUS-12), and other CRI-Adult subscales (all *ps* > 0.05). As a covariate, age was significant for both CRI-Adult positive reappraisal (*F*_(1,49)_ = 8.46, *p* = 0.01, *ηp*^*2*^ = 0.15) and alternative rewards (*F*_(1,49)_ = 5.73, *p* = 0.02, *ηp*^*2*^ = 0.11) (Table 4).

**Table 4:** Gender differences

	Males (*n* = 37)	Females (*n* = 14)			
	M (SD)/*n*	*M* (*SD*)/*n*	*t*_(49)_*/*^*χ2*^	df	*p*
Age	65.38 (10.75)	57.21 (12.18)	5.45	49	0.02
Education	10.85 (3.42)	9.85 (4.18)	0.73	49	0.40
Marital status			0.64	3	0.88
Single/never married	6	2			
Married or living with S/O	26	11			
Divorced/separated	4	1			
Widowed	1	0			
Occupation			12.27	6	0.06
Full-time employed	11	3			
Part-time employed	0	1			
Housewife	0	3			
Unemployed	3	1			
Retired	18	4			
Not able to work for disability	1	0			
Other	4	2			
Disease duration	16.78 (29.39)	20 (37.25)	0.10	49	0.75
Tumor localization			8.20	8	0.41
Salivary glands	4	0			
Pharynx	17	8			
Oral cavity	8	4			
Larynx	5	1			
Paranasal sinus and nasal cavity	2	0			
Skin	2	0			
Tumor stage			0.19	7	10.02
I	5	0			
II	4	0			
III	9	3			
IV	15	9			
Not possible to specify	4	2			
Previous treatments for HNC	17	8	0.51	1	0.47
Esthetic damage	19	10	1.67	1	0.20
Functional damage	29	12	0.35	1	0.56
Stoma	5	1	0.40	1	0.53
SF-12 PCS	43.53 (8.98)	38.31 (5.46)	3.29	49	0.08
SF-12 MCS	46.61 (11.26)	33.52 (8.99)	12.32	49	0.001
DASS-21	13.73 (11.77)	22.85 (8.16)	7.05	49	0.01
Logical analysis (CRI-Adult)	7.52 (4.73)	8.27 (3.27)	0.29	49	0.59
Positive reappraisal (CRI-Adult)	9.92 (4.24)	8.61 (4.27)	3.84	49	0.06
Guidance/support seeking (CRI-Adult)	10.87 (3.43)	11.53 (3.60)	0.36	49	0.55
Problem-solving (CRI-Adult)	11.18 (3.30)	10.08 (3.62)	0.99	49	0.32
Cognitive avoidance (CRI-Adult)	7.42 (2.80)	9.54 (3.45)	4.82	49	0.03
Resigned acceptance (CRI-Adult)	7.94 (3.77)	9.66 (2.59)	2.29	49	0.14
Alternative rewards (CRI-Adult)	6.22 (4.27)	6.38 (4.29)	0.49	49	0.49
Emotional discharge (CRI-Adult)	3.46 (2.74)	6.77 (2.17)	15.47	49	*p* < 0.001
Pain (BPI)	11.76 (13.51)	24.07 (18.37)	6.87	49	0.01
Intolerance of uncertainty (IUS-R)	27.57 (9.39)	31.92 (7.03)	2.30	49	0.14
Social anxiety symptoms (SIAS)	10.88 (7.84)	19.10 (13)	7.64	49	0.01
Self-esteem (RSES)	34.63 (3.57)	29.14 (2.48)	27.80	49	*p* < 0.001
Body image distress (BIS)	4.26 (7.27)	14.23 (7.62)	18.16	49	*p* < 0.001

*Note. M*, mean; *SD*, standard deviation; *df*, degrees of freedom; *S/O*, significant other; *HNC*, head and neck cancer; *SF-12 PCS*, Physical Component Score; *SF-12 MCS*, Mental Component Score; *DASS-21*, Depression Anxiety Stress Scale-21; *CRI-Adult*, Coping Responses Inventory-Adult Form; *BPI*, Brief Pain Inventory; *IUS-R*, Intolerance of Uncertainty Scale-Revised; *SIAS,* Social Interaction Anxiety Scale; *RSES,* Rosenberg Self-Esteem Scale; *BIS,* Body Image Scale. SF-12 *p* value = 0.025; CRI-Adult *p* value = 0.00625

## Discussion

The current research tried to shed light on the relationship between QoL and body image distress in patients with HNC, controlling for relevant psychological variables. Our findings showed that body image distress was negatively associated with physical QoL. The esthetic impairment due to HNC disease is difficult to hide for patients: this might be particularly detrimental in our society, which places great emphasis on attractiveness, leading people to define their identity in terms of physical appearance [47]. When body image distress is high, individuals may fail to engage in healthy behaviors because of a perceived inability to make changes in their physical appearance. This might lead patients to engage in maladaptive behaviors (e.g., smoking) in a desperate attempt to cope with the distress [48], with negative consequences on physical functioning [49]. These findings are in accordance with previous studies among prostate and breast cancer patients (e.g., [50, 51]), which report a similar relationship between body image distress and physical QoL. However, since no research has been carried out on patients with HNC, our study represents a first step to fill in this gap, deepening the understanding of how body image distress affects physical QoL of patients with HNC.

Our study also underlined that the presence of pain was associated with lower mental QoL. About 85% of patients with HNC suffer from pain even before the beginning of cancer treatment [52]: the management of orofacial pain is extremely complicated, as this area of the body is subjected to continuous mechanical stress (i.e., speaking, eating, swallowing, etc.). Consistent with other research, when the pain is continuous and uncontrolled, it has a detrimental effect on every aspect of the patient’s life [53, 54]: (1) interferes with the ability to function; (2) hinders the ability to play social and professional roles; and (3) reduces the person’s independence. Thus, it is not surprising that in our study pain was negatively associated with the mental component of QoL. Since poor QoL is associated with a higher risk of mortality in HNC patients [55–57], and psychological interventions targeting body image distress and pain have shown their effectiveness [58], focusing on these dimensions before HNC treatment might be crucial for improving patients’ overall QoL and survival rates.

Our results also offered important insights with respect to gender differences, showing that female patients with HNC are more impaired than males, despite no gender differences in HNC-related variables emerged. In accordance, women scored higher than males on most of the explored psychological variables and reported lower levels of mental QoL and self-esteem. These results are in line with a previous study [59] pointing out that women report more psychological symptoms than men; this might indicate a gender difference in our society [59]. Indeed, our culture places a great emphasis on females’ physical appearance [60] and attractiveness [61], and women with HNC may perceive a considerable discrepancy between their body and the beauty standards portrayed by society due to appearance changes in the head and neck area, which may negatively impact their body image [2]. Consistently, our results revealed that women experience higher body image distress than males, in accordance with previous studies (e.g., [62, 63]). The higher body image distress in females may also explain the higher psychological distress experienced by females compared to males, in accordance with previous studies [59, 64].

Concerning social anxiety, our results showed higher levels of social anxiety symptoms among females, in accordance with the study by Newell [65]. Individuals who perceive themselves as unattractive and experience dissatisfaction towards their body tend to experience high levels of social anxiety [11], and women in the current sample emerged as characterized by higher body image distress than men. The higher body image distress in women may also explain their lower self-esteem. As emerged in a previous study conducted among cancer patients [66], self-esteem is negatively associated with body image distress in women [67]. Pertaining to coping strategies, women with HNC reported a higher employment of venting of emotions than men. Given that men are less likely to express emotions through venting [68], it is not surprising that females reported higher scores for this coping strategy. These results are consistent with a recent study [69] showing that women with cancer had higher scores of avoidant coping strategies than males. A possible explanation for these findings might be that the employment of venting of emotions may help women with HNC to temporarily distance from negative emotions.

Regarding oncological pain, our results support previous evidence showing that women bear an unequal burden of pain compared to men [7, 70], despite no gender differences in terms of HNC-related variables and physical QoL emerged in the current sample. The higher presence of pain in females might be explained by several factors [71, 72]: (1) influence of sexual hormones (e.g., gonadal hormones); (2) differences in emotional experiences and coping strategies; and (3) differences in social and occupational roles. The higher presence of oncological pain in females might explain their lower mental QoL, in accordance with previous studies [59, 70]. Indeed, pain may be a predisposing factor influencing mental QoL among females with cancer [70].

Lastly, no gender differences in terms of IU emerged. This result might be explained by the cancer illness itself (i.e., frequently unknown disease progression and prognosis, fear of cancer recurrence [15]). Within this context impregnated with constant uncertainty, it is reasonable to assume that both genders are equally characterized by IU.

Despite such interesting results, our research is not free from limitations. First of all, the small number of participants may have affected the accuracy of the results and does not allow the generalization of the results obtained. Therefore, results of the current study should be interpreted with caution. However, HNC is a rare oncological disease, affecting 18 people per 100,000 inhabitants in Italy [73]. Future studies should employ bigger samples to explore the relationship between QoL and body image distress and gender differences among patients with HNC. Second, the current research is a cross-sectional study. This does not allow to establish a causal relationship between the independent variables and QoL, since patients were assessed only on a single occasion (i.e., before undergoing treatment). We focused only on the preoperative period to obtain estimates of body image distress associated with the anticipation of a potentially disfiguring treatment. Previous research supports the influence of preoperative expectations/anticipation of disfigurative HNC treatment on distress, anxiety, coping effectiveness, and post-operative satisfaction [21–24], showing that patients reported elevated levels of body image distress even before HNC treatment [25]. However, the absence of post-treatment measures obliges us to interpret the results with caution, since it was not possible to evaluate the studied variables after the HNC treatment. However, this study allows the identification of possible risk factors to be explored in future studies employing longitudinal designs. In addition to this, other studies should focus on comparing HNC with other oncological diseases that may have a detrimental impact on body image (e.g., colon, bone, breast cancer). This may help to clarify the relationship we observed between body image distress and physical QoL. Finally, the self-report questionnaire we employed to assess QoL in our study (i.e., SF-12) was not specific for patients with HNC, despite it having been used in several studies investigating QoL in this population (i.e., [74–76]). The use of a generic QoL questionnaire represents a limitation of the study because generic QoL questionnaires do not measure severe functional impairments relevant in HNC patients (e.g., feeding and speech difficulties, presence of pain, hyposalivation, and trismus). However, these clinical variables showed associations with poor QoL in HNC patients and might have a negative impact on the social functioning of these patients [77]. In accordance with a recent review [78], future studies should investigate QoL using self-report questionnaires specific to HNC population, such as the University of Washington Quality of Life, version 4 (UW-QOL, [79]).

Despite the abovementioned limitations, the current study is, to our knowledge, the first of its kind, since no previous research has analyzed neither the relationship between body image distress and QoL in HNC while considering relevant psychological variables, nor exhaustively examined gender differences in psychological variables among patients with HNC. Indeed, psychological research in the area of HNC is considered to be in its infancy, and this study provides important insights into psychological variables that might be related to QoL in HNC patients. At the same time, findings of this study may have important clinical implications for early identification and treatment of body image distress and pain in patients with HNC, with the ultimate goal of enhancing the QoL of these patients, guiding the development of a patient-tailored care.

## Supplementary Information

Below is the link to the electronic supplementary material.

Supplementary file1 (DOCX 22.3 KB)

## Data Availability

The data that support the findings of this study are available from the corresponding author upon reasonable request.

## Footnotes

1 The patient received the HNC diagnosis during April 2019 and she was about to receive a surgical treatment with curative intent at the time of the study. In terms of previous diagnosis, she had a history of breast cancer.

2 Given that genders differed in terms of age, Pearson’s correlations were performed to assess the relationship between age and self-report measures. Significant correlations only emerged between age and two subscales of the CRI-Adult: positive reappraisal (*r* =  − 0.32; *p* = 0.02) and alternative rewards (*r* =  − 0.32; *p* = 0.03); therefore, one-way analysis of covariance (ANCOVA) was performed to compare groups only on these CRI-Adult subscales.
