# Supplementary material for: Psychological variables associated with quality of life in patients with head and neck cancer: the role of body image distress
Source: Support Care Cancer. 2022 Aug 23;30(11):9127–39. doi: 10.1007/s00520-022-07334-6 (PMC9633472; doi:10.1007/s00520-022-07334-6)
Supplement: Supplementary file 1 — Supplementary file1 (DOCX 22.3 KB) [file 520_2022_7334_MOESM1_ESM.docx]

Correlations among variables on the whole sample (*n* = 51).

|  | **1** | **2** | **3** | **4** | **5** | **6** | **7** | **8** | **9** | **10** | **11** | **12** | **13** | **14** | **15** | **16** | **17** | **18** |
| --- | --- | --- | --- | --- | --- | --- | --- | --- | --- | --- | --- | --- | --- | --- | --- | --- | --- | --- |
| **1.** SF-12 Physical Composite Score | 1 |  |  |  |  |  |  |  |  |  |  |  |  |  |  |  |  |  |
| **2.** SF-12 Mental Composite Score | .41** | 1 |  |  |  |  |  |  |  |  |  |  |  |  |  |  |  |  |
| **3.** Disease duration | -.34* | -.28 | 1 |  |  |  |  |  |  |  |  |  |  |  |  |  |  |  |
| **4.** Age | -.26 | .02 | -.05 | 1 |  |  |  |  |  |  |  |  |  |  |  |  |  |  |
| **5.** DASS-21 total score | -.40** | -.59** | .27 | .07 | 1 |  |  |  |  |  |  |  |  |  |  |  |  |  |
| **6.** CRI-Adult Logical Analysis | -.15 | -.31* | .11 | -.13 | .41** | 1 |  |  |  |  |  |  |  |  |  |  |  |  |
| **7.** CRI-Adult Positive Reappraisal | .27 | .18 | -.17 | -.32* | -.21 | .26 | 1 |  |  |  |  |  |  |  |  |  |  |  |
| **8.** CRI-Adult Support Seeking | .21 | .07 | -.08 | -.07 | .12 | .27 | .27 | 1 |  |  |  |  |  |  |  |  |  |  |
| **9.** CRI-Adult Problem Solving | .21 | -.14 | .06 | -.07 | .26 | .43** | .41** | .25 | 1 |  |  |  |  |  |  |  |  |  |
| **10.** CRI-Adult Cognitive Avoidance | -.27 | -.26 | .07 | -.23 | .32* | .37** | .04 | .24 | .11 | 1 |  |  |  |  |  |  |  |  |
| **11.** CRI-Adult Resigned Acceptance | -.40** | -.51** | .37** | .02 | .60** | .52** | -.20 | .11 | .05 | .27 | 1 |  |  |  |  |  |  |  |
| **12.** CRI-Adult Alternative Rewards | .34 | .09 | .01 | -.32* | .03 | .10 | .53** | .14 | .41** | .14 | -.12 | 1 |  |  |  |  |  |  |
| **13.** CRI-Adult Emotional Discharge | -.15 | -.46** | .12 | -.20 | .57** | .41** | -.002 | .19 | .03 | -30* | .44* | .20 | 1 |  |  |  |  |  |
| **14.**  BPI total score | -.43** | -.60** | .15 | -.07 | .39** | .31** | -.07 | -.14 | -.02 | .34* | .48** | .04 | .31* | 1 |  |  |  |  |
| **15.** SIAS total score | -.06 | -.29 | -.09 | -.09 | .53** | .11 | -.07 | -.06 | .02 | .28 | .19 | .10 | .58** | .17 | 1 |  |  |  |
| **16.** IUS total score | -.06 | -.17 | -.22 | .14 | .24 | .06 | -.04 | .20 | -.02 | -.02 | .06 | -.01 | .26 | .13 | .02 | 1 |  |  |
| **17.** RSES total score | .42** | .30** | -.02 | .16 | -.44** | -.15 | .17 | -.07 | .21 | -.23 | -.34* | .03 | -.45** | -.26 | -.35** | -.18 | 1 |  |
| **18.** BIS total score | -.52** | -.46** | .32** | -.07 | .49** | .18 | -.19 | .11 | .11 | .35* | .41** | .01 | .36* | .33* | .30* | .19 | -.52** | 1 |

*Note.* * *p* < .05; ** *p* < .001; PCS = Physical Component Score; MCS = Mental Component Score; DASS-21 = Depression Anxiety Stress Scale-21; CRI-Adult: Coping Responses Inventory-Adult Form; BPI = Brief Pain Inventory; SIAS = Social Anxiety Stress Sale; IUS = Intolerance of Uncertainty Scale; RSES = Rosenberg Self-Esteem Scale; BIS = Body Image Scale.
